# Learning biophysically-motivated parameters for alpha helix prediction

**DOI:** 10.1186/1471-2105-8-S5-S3

**Published:** 2007-05-24

**Authors:** Blaise Gassend, Charles W O'Donnell, William Thies, Andrew Lee, Marten van Dijk, Srinivas Devadas

**Affiliations:** 1 Computer Science and Artificial Intelligence Laboratory, Massachusetts Institute of Technology, Cambridge, Massachusetts, USA

## Abstract

**Background:**

Our goal is to develop a state-of-the-art protein secondary structure predictor, with an intuitive and biophysically-motivated energy model. We treat structure prediction as an optimization problem, using parameterizable cost functions representing biological "pseudo-energies". Machine learning methods are applied to estimate the values of the parameters to correctly predict known protein structures.

**Results:**

Focusing on the prediction of alpha helices in proteins, we show that a model with 302 parameters can achieve a Q_*α *_value of 77.6% and an SOV_*α *_value of 73.4%. Such performance numbers are among the best for techniques that do not rely on external databases (such as multiple sequence alignments). Further, it is easier to extract biological significance from a model with so few parameters.

**Conclusion:**

The method presented shows promise for the prediction of protein secondary structure. Biophysically-motivated elementary free-energies can be learned using SVM techniques to construct an energy cost function whose predictive performance rivals state-of-the-art. This method is general and can be extended beyond the all-alpha case described here.

## Background

It remains an important and relevant problem to accurately predict the secondary structure of proteins based on their amino acid sequence. The identification of basic secondary structure elements – alpha helices, beta strands, and coils – is a critical prerequisite for many tertiary structure predictors, which consider the complete three-dimensional protein structure. To date, there has been a broad array of approaches to secondary structure prediction, including statistical techniques, neural networks, hidden Markov models, support vector machines, nearest neighbor methods and energy minimization. In terms of prediction accuracy, neural networks are among the most popular methods in use today [1,2], delivering a pointwise prediction accuracy (Q_3_) of about 77% and a segment overlap measure (SOV) [3] of about 74% [4].

However, to improve the long-term performance of secondary structure prediction, it likely will be necessary to develop a cost model that mirrors the underlying biological constraints. While neural networks offer good performance today, their operation is largely opaque. Often containing up to 10,000 parameters and relying on complex layers of non-linear perceptrons, neural networks offer little insight into the patterns learned. Moreover, they mask the shortcomings of the underlying models, rendering it a tedious and ad-hoc process to improve them. In fact, in the past 15 years, the largest improvements in neural network prediction accuracy have been due to the integration of homologous sequence alignments [4] rather than specific changes to the underlying cost model.

In our approach we focus on simpler, more natural cost models that are based on the underlying biophysics. Due to the lack of experimentally determined free energy values, we begin with parameterizable cost functions, and treat parameter value estimation as an optimization problem. Our goal is then to determine the values of these "pseudo-energies" such that they correctly predict known protein structures. An iterative constraint-based optimization method is used to do this machine learning, incorporating the power of Support Vector Machines (SVMs).

Using a cost function based on Hidden Markov Models (HMMs), we develop a secondary structure predictor for all-alpha proteins. With only 302 parameters, representing the energetic benefit for each residue being in a helix or being a certain distance from the N- or C-cap, our predictor achieves a Q_*α *_value of 77.6% and a SOV_*α *_score of 73.4% when applied to a database of all-alpha proteins. Our technique does not depend on any homologous sequence alignments. When compared to other methods that do not utilize alignment information, it appears that our Q_*α *_represents a 3.5% improvement of the previous best [5], while our SOV_*α *_is comparable (0.2% better). However, due to differences in the data set, we emphasize the novelty of the approach rather than the exact magnitude of the improvements. We are extending our technique to beta strands (and associated data sets) as ongoing work.

### Related work

King and Sternberg share our goal of identifying a small and intuitive set of parameters in the design of the DSC predictor [6]. DSC is largely based on the classic GOR technique [7], which tabulates (during training) the frequency with which each residue appears at a given offset (-8 to +8) from a given structure element (helix, strand, coil). During prediction, each residue is assigned the structure that is most likely given the recorded frequencies for the surrounding residues. King and Sternberg augment the GOR algorithm with several parameters, including the distance to the end of the chain and local patterns of hydrophobicity. They use linear discrimination to derive a statistically favorable weighting of the parameters, resulting in a simple linear cost function; they also perform homologous sequence alignment and minor smoothing and filtering. Using about 1,000 parameters, they estimate an accuracy of *Q*_*α *_= 73.5% for DSC. The primary difference between our predictor and DSC is that we achieve comparable accuracy (our *Q*_*α *_= 77.6%) without providing alignment information. Incorporating an alignment profile is often responsible for 5–7% improvement in accuracy [8-10]. In addition, we learn the position-specific residue affinities rather than using the GOR frequency count. We also consider multiple predictions simultaneously and maintain a global context rather than predicting each residue independently.

Many researchers have developed Hidden Markov Models (HMMs) for secondary structure prediction. Once it has been trained, our predictor could be converted to an HMM without losing any predictive power, as our dynamic programming procedure parallels the Viterbi algorithm for reconstructing the most likely hidden states. However, for the training phase, our system represents a soft-margin Hidden Markov SVM [11] rather than a traditional HMM. Unlike an HMM, a Hidden Markov SVM has a discriminative learning procedure based on a maximum margin criterion and can incorporate "overlapping features", driving the learning based on the overall predicted structure rather than via local propagation.

Tsochantaridis, Altun and Hofmann apply an integrated HMM and SVM framework for secondary structure prediction [12]. The technique may be similar to ours, as we are using their SVM implementation; unfortunately, there are few details published. Nguyen and Rajapakse also present a hybrid scheme in which the output of a Bayesian predictor is further refined by an SVM classifier [13]. The *Q*_*α *_score is 74.1% for the Bayesian predictor alone and 77.0% for the Bayesian/SVM hybrid; the *SOV*_*α *_score is 73.2% for the Bayesian predictor and a comparable 73.0% for the Bayesian/SVM hybrid. To the best of our knowledge, these are the highest *Q*_*α *_and SOV_*α *_scores to date (as tested on Rost and Sander's data set [9]) for a method that does not utilize alignment information.

Bystroff, Thorsson, and Baker design an HMM to recognize specific structural motifs and assemble them into protein secondary structure predictions [14]. Using alignment profiles, they report an overall *Q*_3 _value of 74.3%. Our approach may use fewer parameters, as they manually encode each target motif into a separate set of states. Martin, Gibrat, and Rodolphe develop a 21-state HMM model with 471 parameters that achieves an overall *Q*_3 _value of 65.3% (without alignment profiles) and 72% (with alignment profiles) [15]. Alpha helices are identified based on an amphiphilic motif: a succession of two polar residues and two non-polar residues. Won, Hamelryck, Prügel-Bennet and Krogh give a genetic algorithm that automatically evolves an HMM for secondary structure prediction [16,17]. Using alignment profiles, they report an overall *Q*_3 _value of 75% (only 69.4% for helices). They claim that the resulting 41-state HMM is better than any previous hand-designed HMM. While they restrict their HMM building blocks to "biologically meaningful primitives", it is unclear if there is a natural energetic interpretation of the final HMM.

Schmidler, Liu, and Brutlag develop a segmental semi-Markov Model (a generalization of the HMM), allowing each hidden state to produce a variable-length sequence of the observations [18,19]. They report a *Q*_3 _value of 68.8% without using alignment profiles. Chu and Ghahramani push further in the same direction, merging with the structure of a neural network and demonstrating modest (~1%) improvements over Schmidler et al. [20].

While our technique is currently limited to an alpha helix predictor, for this task it performs better (*Q*_*α *_= 77.6%) than any of the HMM-based methods described above; furthermore, it does so without any alignment information. Our technique is fundamentally different in its use of Hidden Markov SVMs for the learning stage. Lastly, some groups have applied HMM-based predictors to the specific case of transmembrane proteins, where much higher accuracy can be obtained at the expense of generality [21].

There has been a rich and highly successful body of work applying neural networks to secondary structure prediction. The efforts date back to Quian and Sejnowski, who design a simple feed-forward network for the problem [22]. Rost and Sander pioneered the automatic use of multiple sequence alignments to improve the accuracy as part of their PHD predictor [9], which was the top performer at CASP2. More recently, Jones employed the PSI-BLAST tool to efficiently perform the alignments, boosting his PSIPred predictor [4] to the top of CASP3. Baldi and colleagues employ bidirectional recurrent networks in SSPro [23], a system that provided the foundation for Pollastri and McLysaght's Porter server [24].

Petersen describes a ballotting system containing as many as 800 neural networks; while an ensemble of predictors is commonly used to gather more information, this effort is distinguished by its size [25]. A neural network has been followed by an HMM, resulting in a simple and fast system [26]; neural networks have also been used as a post-processing step for GOR predictors [27].

The PSIPred predictor [4] is among the highest scoring neural network techniques. While it achieves an overall *Q*_3 _of about 77% and an SOV of 74%, its performance for alpha helices is even higher: for recent targets on EVA, an open and automatic testing platform [28], PSIPred offers an SOV_*α *_of 78.6% (EVA does not publish a *Q*_*α *_value comparable to ours).

Though state-of-the-art neural network predictors such as PSIPred currently out-perform our method by about 5%, they incorporate multiple sequence alignments and are often impervious to analysis and understanding. In particular, the number of parameters in a neural network can be an order of magnitude higher than that of an HMM-based approach (see Table 1). A notable exception is the network of Riis and Krogh, which is structured by hand to reduce the parameter count to as low as 311 (prediction accuracy is reported at *Q*_3 _= 71.3% with alignment profiles, a good number for its time).

Recently, Support Vector Machines (SVMs) have also been used as a standalone tool for secondary structure prediction [29-34]. In contrast to our technique, which uses an SVM only for learning the parameters of an HMM, these methods apply an SVM directly to a window of residues and classify the central residue into a given secondary structure class. The number of parameters in these techniques depends on the number of support vectors; in one instance, the support vectors occupy 680 MB of memory [30]. Regardless of the number of parameters, it can be difficult to obtain a biological intuition for an SVM, given the non-linear kernel functions and numerous support vectors. Nonetheless, these techniques appear to have significant promise, as Nguyen and Rajapakse report an overall *Q*_3 _of 79.5% and an SOV of 76.3% on the PSIPred database [29].

## Results and discussion

We have applied our method to the problem of all-alpha protein secondary structure prediction. We worked with a set of 300 non-homologous all-alpha proteins taken from EVA's largest sequence-unique subset [35] of the PDB at the end of July 2005. The sequences and structures have been extracted from PDB data processed by DSSP [36]. Only alpha helices have been considered (H residues in DSSP files); everything else has been lumped as *coil *regions.

**Figure 1:**
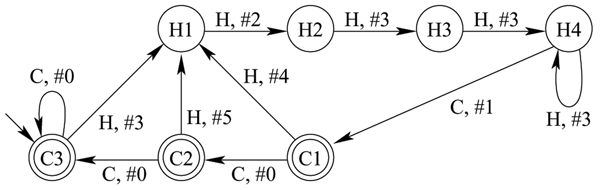
**Predictor finite state machine**. Double circles represent accept states. The arrow leading into state C3 indicates that it is an initial state. Each transition is labeled with the type of structure it corresponds to: helix (H) or coil (C), and a label (#i) indicating which features correspond to this transition in Table 4.

In our experiments, we split our 300 proteins into two 150 protein subsets. The first set is used to train our parameterizable cost function; the second set is used to evaluate the cost function once its parameters have been learned. Since the results vary a bit depending on how the proteins are split in two sets, we train the cost function on 20 random partitions into training and test sets, and report the average performance. Our predictor minimizes the free-energy function *G *using the Viterbi algorithm on a simple 7-state Finite State Machine (shown in Figure 1). The Finite State Machine recognizes alpha helices of length greater than 3 amino acids using 302 elementary free-energies as learned weights. These weigh each amino acid's propensity to be within a helix (20 energies), or within three residues of an N- or C-cap of a helix (20 × 7 × 2 energies). Two weights also penalize 1 and 2 length coils. The motivation for and implementation of the Finite State Machine is described in more detail later.

**Figure 2:**
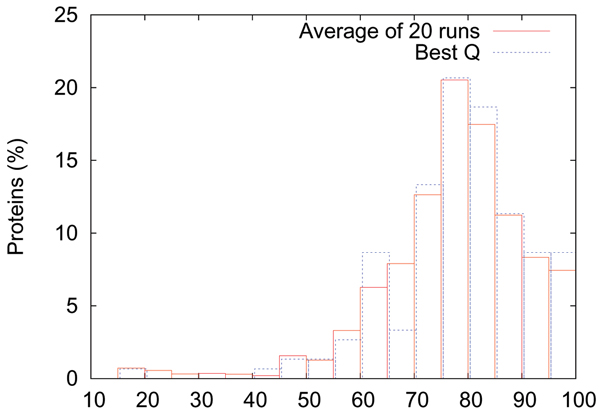
**Q_*α *_accuracy histogram**. Histogram showing the distribution of Q_*α *_across proteins in the test set. We have shown the average case, and the best of the 20 runs which has the highest Q_*α*_.

**Figure 3:**
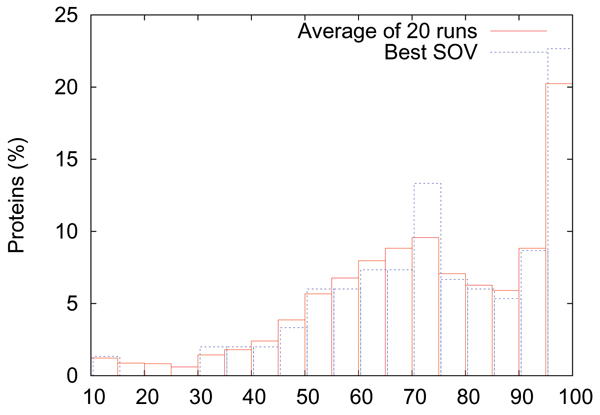
**SOV_*α *_accuracy histogram**. Histogram showing the distribution of SOV_*α *_across proteins in the test set. We have shown the average case, and the best of the 20 runs which has the highest SOV_*α*_.

Table 2 presents our total results using both the Q_*α *_and SOV_*α *_metrics. Figures 2 and 3 show histograms detailing the distribution of each score. The Q_*α *_metric is simply the number of correctly predicted residues divided by sequence length. SOV_*α *_is a more elaborate metric that has been designed to ignore small errors in helix-coil transition position, but heavily penalize more fundamental errors such as gaps appearing in a helix [3].

On average, our method predicts helices in all-alpha proteins with an accuracy of 77.6% (Q_*α*_) or 73.4% (SOV_*α*_). Unfortunately, these results are difficult to compare with existing prediction methods which usually do predictions on both alpha helices and beta strands. Rost and Sanders caution that restricting the test set to all-alpha proteins can result in up to a 3% gain in accuracy [9]. Nonetheless, if one does compare our technique with the previous best amongst methods that do not utilize alignment information [5], our results represent a 3.5% improvement in Q_*α *_and a 0.2% improvement in SOV_*α*_.

Additional care should be taken in comparing these numbers to recent techniques such as PSIPred [4], which consider 3_10 _helices (the DSSP state 'G') to be part of a helix rather than a loop; they report gains of about 2% in overall *Q*_3 _if helices are restricted to 4-helices (as in most HMM techniques, including ours). Apart from prediction accuracy, our technique is distinguished from others by its emphasis on an intuitive and biophysically-motivated cost function. While some of techniques require upwards of 10,000 parameters (see Table 1), our predictor achieves competitive accuracy using only 302 parameters.

The real power of the machine learning method we use is its applicability beyond HMM models. As will become evident in the description of the method, we could describe protein structures as a parse tree of a context-free grammar (or multi-tape grammar) rather than as a sequence of HMM states. With these enriched descriptions, we should be able to include in the cost function interactions between adjacent strands of a beta sheet. This should allow us to incorporate beta sheet prediction into our algorithm.

Unlike most secondary structure methods, we would then be able to predict not only which residues participate in a beta sheet, but also which residues are forming hydrogen bonds between adjacent sheets.

## Conclusion

This work is a promising first pass at using SVM techniques to find the elementary free-energies needed to predict protein secondary structure. The method we use is general and can be extended beyond the all-alpha case described here. In future work, we plan to extend this method to super-secondary structure prediction, generating contact maps of individual hydrogen bonds in beta sheets.

## Methods

It is widely believed that when a protein is folded, its free-energy approaches a thermodynamic minimum. We therefore treat structure prediction as an optimization problem.

### Formal optimization problem

In our technique, we define a free-energy function *G*(**x**, **y**) that estimates the free-energy of an amino acid sequence **x **when folded into a candidate secondary structure **y**. Our predictor outputs the secondary structure y^
 MathType@MTEF@5@5@+=feaafiart1ev1aaatCvAUfKttLearuWrP9MDH5MBPbIqV92AaeXatLxBI9gBaebbnrfifHhDYfgasaacH8akY=wiFfYdH8Gipec8Eeeu0xXdbba9frFj0=OqFfea0dXdd9vqai=hGuQ8kuc9pgc9s8qqaq=dirpe0xb9q8qiLsFr0=vr0=vr0dc8meaabaqaciaacaGaaeqabaqabeGadaaakeaaieqacuWF5bqEgaqcaaaa@2E3D@ that has the minimal free-energy according to *G*:

y^=arg⁡min⁡y∈YG(x,y).     (1)
 MathType@MTEF@5@5@+=feaafiart1ev1aaatCvAUfKttLearuWrP9MDH5MBPbIqV92AaeXatLxBI9gBaebbnrfifHhDYfgasaacH8akY=wiFfYdH8Gipec8Eeeu0xXdbba9frFj0=OqFfea0dXdd9vqai=hGuQ8kuc9pgc9s8qqaq=dirpe0xb9q8qiLsFr0=vr0=vr0dc8meaabaqaciaacaGaaeqabaqabeGadaaakeaaieqacuWF5bqEgaqcaiabg2da9maaxababaGagiyyaeMaeiOCaiNaei4zaCMagiyBa0MaeiyAaKMaeiOBa4galeaacqWF5bqEcqGHiiIZt0uy0HwzTfgDPnwy1egaryqtHrhAL1wy0L2yHvdaiqaacqGFyeFwaeqaaOGaem4raCKaeiikaGIae8hEaGNaeiilaWIae8xEaKNaeiykaKIaeiOla4IaaCzcaiaaxMaadaqadaqaaiabigdaXaGaayjkaiaawMcaaaaa@518A@

To go from this general statement to a working algorithm, we need to a find free-energy function *G *and a set of structures Y
 MathType@MTEF@5@5@+=feaafiart1ev1aaatCvAUfKttLearuWrP9MDH5MBPbIqV92AaeXatLxBI9gBaebbnrfifHhDYfgasaacH8akY=wiFfYdH8Gipec8Eeeu0xXdbba9frFj0=OqFfea0dXdd9vqai=hGuQ8kuc9pgc9s8qqaq=dirpe0xb9q8qiLsFr0=vr0=vr0dc8meaabaqaciaacaGaaeqabaqabeGadaaakeaat0uy0HwzTfgDPnwy1egaryqtHrhAL1wy0L2yHvdaiqaacqWFyeFwaaa@3850@ for which the minimization shown in equation (1) is easy to compute. In choosing *G *and Y
 MathType@MTEF@5@5@+=feaafiart1ev1aaatCvAUfKttLearuWrP9MDH5MBPbIqV92AaeXatLxBI9gBaebbnrfifHhDYfgasaacH8akY=wiFfYdH8Gipec8Eeeu0xXdbba9frFj0=OqFfea0dXdd9vqai=hGuQ8kuc9pgc9s8qqaq=dirpe0xb9q8qiLsFr0=vr0=vr0dc8meaabaqaciaacaGaaeqabaqabeGadaaakeaat0uy0HwzTfgDPnwy1egaryqtHrhAL1wy0L2yHvdaiqaacqWFyeFwaaa@3850@, we tradeoff the ability to efficiently minimize *G *with the ability to accurately capture the richness and detailed physics of protein structure. Atomistic models are able to capture the whole range of structures, and incorporate all the physical interactions between atoms. However, because of this detail they can only be optimized using heuristic methods. We therefore prefer to consider a simplified set of structures Y
 MathType@MTEF@5@5@+=feaafiart1ev1aaatCvAUfKttLearuWrP9MDH5MBPbIqV92AaeXatLxBI9gBaebbnrfifHhDYfgasaacH8akY=wiFfYdH8Gipec8Eeeu0xXdbba9frFj0=OqFfea0dXdd9vqai=hGuQ8kuc9pgc9s8qqaq=dirpe0xb9q8qiLsFr0=vr0=vr0dc8meaabaqaciaacaGaaeqabaqabeGadaaakeaat0uy0HwzTfgDPnwy1egaryqtHrhAL1wy0L2yHvdaiqaacqWFyeFwaaa@3850@, and a cost function *G *with lumped parameters that try to approach physical reality.

These lumped parameters are difficult to determine experimentally. We will therefore define a class G
 MathType@MTEF@5@5@+=feaafiart1ev1aaatCvAUfKttLearuWrP9MDH5MBPbIqV92AaeXatLxBI9gBaebbnrfifHhDYfgasaacH8akY=wiFfYdH8Gipec8Eeeu0xXdbba9frFj0=OqFfea0dXdd9vqai=hGuQ8kuc9pgc9s8qqaq=dirpe0xb9q8qiLsFr0=vr0=vr0dc8meaabaqaciaacaGaaeqabaqabeGadaaakeaat0uy0HwzTfgDPnwy1egaryqtHrhAL1wy0L2yHvdaiqaacqWFge=raaa@382C@ of candidate free-energy functions that are easy to optimize over some set of structures Y
 MathType@MTEF@5@5@+=feaafiart1ev1aaatCvAUfKttLearuWrP9MDH5MBPbIqV92AaeXatLxBI9gBaebbnrfifHhDYfgasaacH8akY=wiFfYdH8Gipec8Eeeu0xXdbba9frFj0=OqFfea0dXdd9vqai=hGuQ8kuc9pgc9s8qqaq=dirpe0xb9q8qiLsFr0=vr0=vr0dc8meaabaqaciaacaGaaeqabaqabeGadaaakeaat0uy0HwzTfgDPnwy1egaryqtHrhAL1wy0L2yHvdaiqaacqWFyeFwaaa@3850@. Then we will use machine learning techniques to pick a good *G *from all the candidates in G
 MathType@MTEF@5@5@+=feaafiart1ev1aaatCvAUfKttLearuWrP9MDH5MBPbIqV92AaeXatLxBI9gBaebbnrfifHhDYfgasaacH8akY=wiFfYdH8Gipec8Eeeu0xXdbba9frFj0=OqFfea0dXdd9vqai=hGuQ8kuc9pgc9s8qqaq=dirpe0xb9q8qiLsFr0=vr0=vr0dc8meaabaqaciaacaGaaeqabaqabeGadaaakeaat0uy0HwzTfgDPnwy1egaryqtHrhAL1wy0L2yHvdaiqaacqWFge=raaa@382C@. The machine learning will use structure information from the Protein Data Bank (PDB) [37] to determine which *G *to pick. Given a set of training examples {(**x**_*i*_, **y**_*i*_): *i *= 1,...,*k*}, the learning algorithm needs to find a *G *∈ G
 MathType@MTEF@5@5@+=feaafiart1ev1aaatCvAUfKttLearuWrP9MDH5MBPbIqV92AaeXatLxBI9gBaebbnrfifHhDYfgasaacH8akY=wiFfYdH8Gipec8Eeeu0xXdbba9frFj0=OqFfea0dXdd9vqai=hGuQ8kuc9pgc9s8qqaq=dirpe0xb9q8qiLsFr0=vr0=vr0dc8meaabaqaciaacaGaaeqabaqabeGadaaakeaat0uy0HwzTfgDPnwy1egaryqtHrhAL1wy0L2yHvdaiqaacqWFge=raaa@382C@ such that:

∀i:yi=arg⁡min⁡y∈YG(xi,y).     (2)
 MathType@MTEF@5@5@+=feaafiart1ev1aaatCvAUfKttLearuWrP9MDH5MBPbIqV92AaeXatLxBI9gBaebbnrfifHhDYfgasaacH8akY=wiFfYdH8Gipec8Eeeu0xXdbba9frFj0=OqFfea0dXdd9vqai=hGuQ8kuc9pgc9s8qqaq=dirpe0xb9q8qiLsFr0=vr0=vr0dc8meaabaqaciaacaGaaeqabaqabeGadaaakeaacqGHaiIicqWGPbqAcqGG6aGoieqacqWF5bqEdaWgaaWcbaGaemyAaKgabeaakiabg2da9maaxababaGagiyyaeMaeiOCaiNaei4zaCMagiyBa0MaeiyAaKMaeiOBa4galeaacqWF5bqEcqGHiiIZt0uy0HwzTfgDPnwy1egaryqtHrhAL1wy0L2yHvdaiqaacqGFyeFwaeqaaOGaem4raCKaeiikaGIae8hEaG3aaSbaaSqaaiabdMgaPbqabaGccqGGSaalcqWF5bqEcqGGPaqkcqGGUaGlcaWLjaGaaCzcamaabmaabaGaeGOmaidacaGLOaGaayzkaaaaaa@57C5@

In practice, this *G *may not exist or may not be unique, so the machine learning algorithm may have to pick a good approximation, or select a *G *that is more likely to generalize well to proteins not in the training set. We will now look more closely at how a good *G *is selected and later we will be more specific about what G
 MathType@MTEF@5@5@+=feaafiart1ev1aaatCvAUfKttLearuWrP9MDH5MBPbIqV92AaeXatLxBI9gBaebbnrfifHhDYfgasaacH8akY=wiFfYdH8Gipec8Eeeu0xXdbba9frFj0=OqFfea0dXdd9vqai=hGuQ8kuc9pgc9s8qqaq=dirpe0xb9q8qiLsFr0=vr0=vr0dc8meaabaqaciaacaGaaeqabaqabeGadaaakeaat0uy0HwzTfgDPnwy1egaryqtHrhAL1wy0L2yHvdaiqaacqWFge=raaa@382C@ and Y
 MathType@MTEF@5@5@+=feaafiart1ev1aaatCvAUfKttLearuWrP9MDH5MBPbIqV92AaeXatLxBI9gBaebbnrfifHhDYfgasaacH8akY=wiFfYdH8Gipec8Eeeu0xXdbba9frFj0=OqFfea0dXdd9vqai=hGuQ8kuc9pgc9s8qqaq=dirpe0xb9q8qiLsFr0=vr0=vr0dc8meaabaqaciaacaGaaeqabaqabeGadaaakeaat0uy0HwzTfgDPnwy1egaryqtHrhAL1wy0L2yHvdaiqaacqWFyeFwaaa@3850@ are.

### Iterative constraint based approach

First, we notice that equation (2) can be rewritten as the problem of finding a function *G *that satisfies the large set of inequality constraints

∀*i*, ∀*y *∈ Y
 MathType@MTEF@5@5@+=feaafiart1ev1aaatCvAUfKttLearuWrP9MDH5MBPbIqV92AaeXatLxBI9gBaebbnrfifHhDYfgasaacH8akY=wiFfYdH8Gipec8Eeeu0xXdbba9frFj0=OqFfea0dXdd9vqai=hGuQ8kuc9pgc9s8qqaq=dirpe0xb9q8qiLsFr0=vr0=vr0dc8meaabaqaciaacaGaaeqabaqabeGadaaakeaat0uy0HwzTfgDPnwy1egaryqtHrhAL1wy0L2yHvdaiqaacqWFyeFwaaa@3850@\{**y**_*i*_}: *G*(**x**_*i*_, **y**_*i*_) <*G*(**x**_*i*_, **y**).     (3)

Unfortunately, the set of all secondary structures Y
 MathType@MTEF@5@5@+=feaafiart1ev1aaatCvAUfKttLearuWrP9MDH5MBPbIqV92AaeXatLxBI9gBaebbnrfifHhDYfgasaacH8akY=wiFfYdH8Gipec8Eeeu0xXdbba9frFj0=OqFfea0dXdd9vqai=hGuQ8kuc9pgc9s8qqaq=dirpe0xb9q8qiLsFr0=vr0=vr0dc8meaabaqaciaacaGaaeqabaqabeGadaaakeaat0uy0HwzTfgDPnwy1egaryqtHrhAL1wy0L2yHvdaiqaacqWFyeFwaaa@3850@ is exponentially large, so finding a *G *∈ G
 MathType@MTEF@5@5@+=feaafiart1ev1aaatCvAUfKttLearuWrP9MDH5MBPbIqV92AaeXatLxBI9gBaebbnrfifHhDYfgasaacH8akY=wiFfYdH8Gipec8Eeeu0xXdbba9frFj0=OqFfea0dXdd9vqai=hGuQ8kuc9pgc9s8qqaq=dirpe0xb9q8qiLsFr0=vr0=vr0dc8meaabaqaciaacaGaaeqabaqabeGadaaakeaat0uy0HwzTfgDPnwy1egaryqtHrhAL1wy0L2yHvdaiqaacqWFge=raaa@382C@ that satisfies all these inequalities directly is computationally intractable. Our approach reduces the problem by ignoring as many constraints as possible, only considering the constraints it is "forced" to consider.

In our method, the reduced problem is defined as the problem of finding a function *G' *that satisfies the set of constraints

∀*i*, ∀*y *∈ *S*_*i*_: *G'*(**x**_*i*_, **y**_*i*_) <*G'*(**x**_*i*_, **y**),     (4)

for some *S*_*i *_⊆ Y
 MathType@MTEF@5@5@+=feaafiart1ev1aaatCvAUfKttLearuWrP9MDH5MBPbIqV92AaeXatLxBI9gBaebbnrfifHhDYfgasaacH8akY=wiFfYdH8Gipec8Eeeu0xXdbba9frFj0=OqFfea0dXdd9vqai=hGuQ8kuc9pgc9s8qqaq=dirpe0xb9q8qiLsFr0=vr0=vr0dc8meaabaqaciaacaGaaeqabaqabeGadaaakeaat0uy0HwzTfgDPnwy1egaryqtHrhAL1wy0L2yHvdaiqaacqWFyeFwaaa@3850@\{**y**_*i*_}.

Initially, we begin with no constraints at all (that is, *S*_*i *_= ∅ for all *i*) and we choose some function *G*' ∈ G
 MathType@MTEF@5@5@+=feaafiart1ev1aaatCvAUfKttLearuWrP9MDH5MBPbIqV92AaeXatLxBI9gBaebbnrfifHhDYfgasaacH8akY=wiFfYdH8Gipec8Eeeu0xXdbba9frFj0=OqFfea0dXdd9vqai=hGuQ8kuc9pgc9s8qqaq=dirpe0xb9q8qiLsFr0=vr0=vr0dc8meaabaqaciaacaGaaeqabaqabeGadaaakeaat0uy0HwzTfgDPnwy1egaryqtHrhAL1wy0L2yHvdaiqaacqWFge=raaa@382C@. Note that, since we start with no constraints, any function *G*' ∈ G
 MathType@MTEF@5@5@+=feaafiart1ev1aaatCvAUfKttLearuWrP9MDH5MBPbIqV92AaeXatLxBI9gBaebbnrfifHhDYfgasaacH8akY=wiFfYdH8Gipec8Eeeu0xXdbba9frFj0=OqFfea0dXdd9vqai=hGuQ8kuc9pgc9s8qqaq=dirpe0xb9q8qiLsFr0=vr0=vr0dc8meaabaqaciaacaGaaeqabaqabeGadaaakeaat0uy0HwzTfgDPnwy1egaryqtHrhAL1wy0L2yHvdaiqaacqWFge=raaa@382C@ initially satisfies equation (4). We then need to check whether *G' *approximates the solution *G *to the set of constraints (2). In particular, we verify whether *G' *can be used to approximate **y**_1 _as the solution y^
 MathType@MTEF@5@5@+=feaafiart1ev1aaatCvAUfKttLearuWrP9MDH5MBPbIqV92AaeXatLxBI9gBaebbnrfifHhDYfgasaacH8akY=wiFfYdH8Gipec8Eeeu0xXdbba9frFj0=OqFfea0dXdd9vqai=hGuQ8kuc9pgc9s8qqaq=dirpe0xb9q8qiLsFr0=vr0=vr0dc8meaabaqaciaacaGaaeqabaqabeGadaaakeaaieqacuWF5bqEgaqcaaaa@2E3D@_1 _of the problem

y^1=arg⁡min⁡y∈YG′(x1,y).
 MathType@MTEF@5@5@+=feaafiart1ev1aaatCvAUfKttLearuWrP9MDH5MBPbIqV92AaeXatLxBI9gBaebbnrfifHhDYfgasaacH8akY=wiFfYdH8Gipec8Eeeu0xXdbba9frFj0=OqFfea0dXdd9vqai=hGuQ8kuc9pgc9s8qqaq=dirpe0xb9q8qiLsFr0=vr0=vr0dc8meaabaqaciaacaGaaeqabaqabeGadaaakeaaieqacuWF5bqEgaqcamaaBaaaleaacqaIXaqmaeqaaOGaeyypa0ZaaCbeaeaacyGGHbqycqGGYbGCcqGGNbWzcyGGTbqBcqGGPbqAcqGGUbGBaSqaaiab=Lha5jabgIGioprtHrhAL1wy0L2yHvtyaeHbnfgDOvwBHrxAJfwnaGabaiab+Hr8zbqabaGccuWGhbWrgaqbaiabcIcaOiab=Hha4naaBaaaleaacqaIXaqmaeqaaOGaeiilaWIae8xEaKNaeiykaKIaeiOla4caaa@5025@

If *G'*(**x**_1_, **y**_1_) <*G'*(**x**_1_, y^
 MathType@MTEF@5@5@+=feaafiart1ev1aaatCvAUfKttLearuWrP9MDH5MBPbIqV92AaeXatLxBI9gBaebbnrfifHhDYfgasaacH8akY=wiFfYdH8Gipec8Eeeu0xXdbba9frFj0=OqFfea0dXdd9vqai=hGuQ8kuc9pgc9s8qqaq=dirpe0xb9q8qiLsFr0=vr0=vr0dc8meaabaqaciaacaGaaeqabaqabeGadaaakeaaieqacuWF5bqEgaqcaaaa@2E3D@_1_) + *ε*, we say that y^
 MathType@MTEF@5@5@+=feaafiart1ev1aaatCvAUfKttLearuWrP9MDH5MBPbIqV92AaeXatLxBI9gBaebbnrfifHhDYfgasaacH8akY=wiFfYdH8Gipec8Eeeu0xXdbba9frFj0=OqFfea0dXdd9vqai=hGuQ8kuc9pgc9s8qqaq=dirpe0xb9q8qiLsFr0=vr0=vr0dc8meaabaqaciaacaGaaeqabaqabeGadaaakeaaieqacuWF5bqEgaqcaaaa@2E3D@_1 _is "close" to **y**_1 _in the sense that y^
 MathType@MTEF@5@5@+=feaafiart1ev1aaatCvAUfKttLearuWrP9MDH5MBPbIqV92AaeXatLxBI9gBaebbnrfifHhDYfgasaacH8akY=wiFfYdH8Gipec8Eeeu0xXdbba9frFj0=OqFfea0dXdd9vqai=hGuQ8kuc9pgc9s8qqaq=dirpe0xb9q8qiLsFr0=vr0=vr0dc8meaabaqaciaacaGaaeqabaqabeGadaaakeaaieqacuWF5bqEgaqcaaaa@2E3D@_1 _is a close enough approximation of **y**_1_. If y^
 MathType@MTEF@5@5@+=feaafiart1ev1aaatCvAUfKttLearuWrP9MDH5MBPbIqV92AaeXatLxBI9gBaebbnrfifHhDYfgasaacH8akY=wiFfYdH8Gipec8Eeeu0xXdbba9frFj0=OqFfea0dXdd9vqai=hGuQ8kuc9pgc9s8qqaq=dirpe0xb9q8qiLsFr0=vr0=vr0dc8meaabaqaciaacaGaaeqabaqabeGadaaakeaaieqacuWF5bqEgaqcaaaa@2E3D@_1 _is close to **y**_1_, we go on to the next optimization problem

y^2=arg⁡min⁡y∈YG′(x2,y).
 MathType@MTEF@5@5@+=feaafiart1ev1aaatCvAUfKttLearuWrP9MDH5MBPbIqV92AaeXatLxBI9gBaebbnrfifHhDYfgasaacH8akY=wiFfYdH8Gipec8Eeeu0xXdbba9frFj0=OqFfea0dXdd9vqai=hGuQ8kuc9pgc9s8qqaq=dirpe0xb9q8qiLsFr0=vr0=vr0dc8meaabaqaciaacaGaaeqabaqabeGadaaakeaaieqacuWF5bqEgaqcamaaBaaaleaacqaIYaGmaeqaaOGaeyypa0ZaaCbeaeaacyGGHbqycqGGYbGCcqGGNbWzcyGGTbqBcqGGPbqAcqGGUbGBaSqaaiab=Lha5jabgIGioprtHrhAL1wy0L2yHvtyaeHbnfgDOvwBHrxAJfwnaGabaiab+Hr8zbqabaGccuWGhbWrgaqbaiabcIcaOiab=Hha4naaBaaaleaacqaIYaGmaeqaaOGaeiilaWIae8xEaKNaeiykaKIaeiOla4caaa@5029@

If y^
 MathType@MTEF@5@5@+=feaafiart1ev1aaatCvAUfKttLearuWrP9MDH5MBPbIqV92AaeXatLxBI9gBaebbnrfifHhDYfgasaacH8akY=wiFfYdH8Gipec8Eeeu0xXdbba9frFj0=OqFfea0dXdd9vqai=hGuQ8kuc9pgc9s8qqaq=dirpe0xb9q8qiLsFr0=vr0=vr0dc8meaabaqaciaacaGaaeqabaqabeGadaaakeaaieqacuWF5bqEgaqcaaaa@2E3D@_1 _is not close to **y**_1_, this means the constraint *G'*(**x**_1_, **y**_1_) <*G'*(**x**_1_, y^
 MathType@MTEF@5@5@+=feaafiart1ev1aaatCvAUfKttLearuWrP9MDH5MBPbIqV92AaeXatLxBI9gBaebbnrfifHhDYfgasaacH8akY=wiFfYdH8Gipec8Eeeu0xXdbba9frFj0=OqFfea0dXdd9vqai=hGuQ8kuc9pgc9s8qqaq=dirpe0xb9q8qiLsFr0=vr0=vr0dc8meaabaqaciaacaGaaeqabaqabeGadaaakeaaieqacuWF5bqEgaqcaaaa@2E3D@_1_) in equation (3) has been violated.

Therefore we must add this constraint to our reduced problem, replacing *S*_1 _by *S*_1 _∪ {y^
 MathType@MTEF@5@5@+=feaafiart1ev1aaatCvAUfKttLearuWrP9MDH5MBPbIqV92AaeXatLxBI9gBaebbnrfifHhDYfgasaacH8akY=wiFfYdH8Gipec8Eeeu0xXdbba9frFj0=OqFfea0dXdd9vqai=hGuQ8kuc9pgc9s8qqaq=dirpe0xb9q8qiLsFr0=vr0=vr0dc8meaabaqaciaacaGaaeqabaqabeGadaaakeaaieqacuWF5bqEgaqcaaaa@2E3D@_1_}. In order to solve the new reduced problem we need to find a new *G' *that satisfies the old and new constraints. At all times the number of constraints in the reduced problem is relatively small such that it is computationally feasible to find its solution.

**Figure 4:**
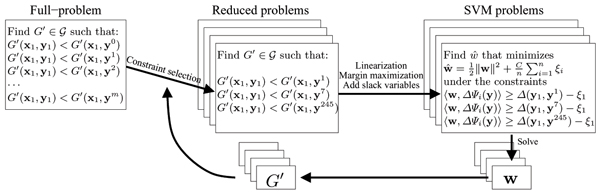
**Summary of learning algorithm**. In this figure each large frame represents a problem that needs to be solved. On the left, we start with an intractably large problem. At each iteration, we pick a subset of the large problem to work on, solve it approximately using an SVM formulation, and use the resulting solution to expand the subset of constraints we are working with.

Whenever a prediction y^
 MathType@MTEF@5@5@+=feaafiart1ev1aaatCvAUfKttLearuWrP9MDH5MBPbIqV92AaeXatLxBI9gBaebbnrfifHhDYfgasaacH8akY=wiFfYdH8Gipec8Eeeu0xXdbba9frFj0=OqFfea0dXdd9vqai=hGuQ8kuc9pgc9s8qqaq=dirpe0xb9q8qiLsFr0=vr0=vr0dc8meaabaqaciaacaGaaeqabaqabeGadaaakeaaieqacuWF5bqEgaqcaaaa@2E3D@_*i *_is not satisfactorily close to **y**_*i*_, we add more constraints. For instance, Figure 4 shows our problem reduction for the training example (**x**_1_, **y**_1_). Note that the reduced problems lead to the constraints *G'*(**x**_1_, **y**_1_) <*G'*(**x**_1_, **y**^1^), *G'*(**x**_1_, **y**_1_) <*G'*(**x**_1_, **y**^7^), *G'*(**x**_1_, **y**_1_) <*G'*(**x**_1_, **y**^245^), etc., where Y
 MathType@MTEF@5@5@+=feaafiart1ev1aaatCvAUfKttLearuWrP9MDH5MBPbIqV92AaeXatLxBI9gBaebbnrfifHhDYfgasaacH8akY=wiFfYdH8Gipec8Eeeu0xXdbba9frFj0=OqFfea0dXdd9vqai=hGuQ8kuc9pgc9s8qqaq=dirpe0xb9q8qiLsFr0=vr0=vr0dc8meaabaqaciaacaGaaeqabaqabeGadaaakeaat0uy0HwzTfgDPnwy1egaryqtHrhAL1wy0L2yHvdaiqaacqWFyeFwaaa@3850@ = {**y**^1^, **y**^2^,...,**y**^*m*^} (in other words, *S*_1 _= {**y**^1^, **y**^7^, **y**^245^}).

The algorithm terminates if no constraints need to be added. That is, each prediction is a good approximation,

∀i:G′(xi,yi)<G′(xi,y^i)+εwhere y^i=arg⁡min⁡y∈YG′(xi,y).     (5)
 MathType@MTEF@5@5@+=feaafiart1ev1aaatCvAUfKttLearuWrP9MDH5MBPbIqV92AaeXatLxBI9gBaebbnrfifHhDYfgasaacH8akY=wiFfYdH8Gipec8Eeeu0xXdbba9frFj0=OqFfea0dXdd9vqai=hGuQ8kuc9pgc9s8qqaq=dirpe0xb9q8qiLsFr0=vr0=vr0dc8meaabaqaciaacaGaaeqabaqabeGadaaakeaacqGHaiIicqWGPbqAcqGG6aGocuWGhbWrgaqbaiabcIcaOGqabiab=Hha4naaBaaaleaacqWGPbqAaeqaaOGaeiilaWIae8xEaK3aaSbaaSqaaiabdMgaPbqabaGccqGGPaqkcqGH8aapcuWGhbWrgaqbaiabcIcaOiab=Hha4naaBaaaleaacqWGPbqAaeqaaOGaeiilaWIaf8xEaKNbaKaadaWgaaWcbaGaemyAaKgabeaakiabcMcaPiabgUcaRGGaciab+v7aLjab+bcaGiabbEha3jabbIgaOjabbwgaLjabbkhaYjabbwgaLjabbccaGiqb=Lha5zaajaWaaSbaaSqaaiabdMgaPbqabaGccqGH9aqpdaWfqaqaaiGbcggaHjabckhaYjabcEgaNjGbc2gaTjabcMgaPjabc6gaUbWcbaGae8xEaKNaeyicI48enfgDOvwBHrxAJfwnHbqeg0uy0HwzTfgDPnwy1aaceaGae0hgXNfabeaakiqbdEeahzaafaGaeiikaGIae8hEaG3aaSbaaSqaaiabdMgaPbqabaGccqGGSaalcqWF5bqEcqGGPaqkcqGGUaGlcaWLjaGaaCzcamaabmaabaGaeGynaudacaGLOaGaayzkaaaaaa@7773@

This is equivalent to

∀*i*, ∀*y *∈ Y
 MathType@MTEF@5@5@+=feaafiart1ev1aaatCvAUfKttLearuWrP9MDH5MBPbIqV92AaeXatLxBI9gBaebbnrfifHhDYfgasaacH8akY=wiFfYdH8Gipec8Eeeu0xXdbba9frFj0=OqFfea0dXdd9vqai=hGuQ8kuc9pgc9s8qqaq=dirpe0xb9q8qiLsFr0=vr0=vr0dc8meaabaqaciaacaGaaeqabaqabeGadaaakeaat0uy0HwzTfgDPnwy1egaryqtHrhAL1wy0L2yHvdaiqaacqWFyeFwaaa@3850@\{**y**_*i*_}:*G'*(**x**_*i*_, **y**_*i*_) <*G'*(**x**_*i*_, **y**) + *ε*.     (6)

This is similar to the full set of constraints on *G *in equation (3), except that *G' *need only satisfy each inequality within a distance of *ε*.

### Linear cost function

One important assumption we make is that the family of free energy functions G
 MathType@MTEF@5@5@+=feaafiart1ev1aaatCvAUfKttLearuWrP9MDH5MBPbIqV92AaeXatLxBI9gBaebbnrfifHhDYfgasaacH8akY=wiFfYdH8Gipec8Eeeu0xXdbba9frFj0=OqFfea0dXdd9vqai=hGuQ8kuc9pgc9s8qqaq=dirpe0xb9q8qiLsFr0=vr0=vr0dc8meaabaqaciaacaGaaeqabaqabeGadaaakeaat0uy0HwzTfgDPnwy1egaryqtHrhAL1wy0L2yHvdaiqaacqWFge=raaa@382C@ is linear. That is, the total free energy of the protein is a sum of elementary interactions. This simplification agrees with many mathematical models of the energy force fields that control protein folding. For example, electrostatic, Van der Waals, stretch, bend, and torsion forces can all be described by the sum of energy terms for each pair of molecular elements. Given this, we can formally define the family of functions G
 MathType@MTEF@5@5@+=feaafiart1ev1aaatCvAUfKttLearuWrP9MDH5MBPbIqV92AaeXatLxBI9gBaebbnrfifHhDYfgasaacH8akY=wiFfYdH8Gipec8Eeeu0xXdbba9frFj0=OqFfea0dXdd9vqai=hGuQ8kuc9pgc9s8qqaq=dirpe0xb9q8qiLsFr0=vr0=vr0dc8meaabaqaciaacaGaaeqabaqabeGadaaakeaat0uy0HwzTfgDPnwy1egaryqtHrhAL1wy0L2yHvdaiqaacqWFge=raaa@382C@ to be

G
 MathType@MTEF@5@5@+=feaafiart1ev1aaatCvAUfKttLearuWrP9MDH5MBPbIqV92AaeXatLxBI9gBaebbnrfifHhDYfgasaacH8akY=wiFfYdH8Gipec8Eeeu0xXdbba9frFj0=OqFfea0dXdd9vqai=hGuQ8kuc9pgc9s8qqaq=dirpe0xb9q8qiLsFr0=vr0=vr0dc8meaabaqaciaacaGaaeqabaqabeGadaaakeaat0uy0HwzTfgDPnwy1egaryqtHrhAL1wy0L2yHvdaiqaacqWFge=raaa@382C@ = {*G*_**w**_: (**x**, **y**) → ⟨**w**, Ψ(**x**, **y**)⟩ : for some **w**}.     (7)

Here the feature function Ψ is fixed and known, representing the specific energy characteristics that we are interested in. For example, one element of the vector Ψ(**x**, **y**) might be the number of proline residues from sequence **x **that appear within an alpha helix in candidate structure **y**. Additional details on our design of Ψ appears later. By definition of a linear function, the dot product of the vector **w **(notated by ⟨, ⟩) can then be taken to appropriately weight the importance of individual terms within Ψ. With this assumption, the reduced problem's constraints given by equation (4) can be rewritten as

∀*i*, ∀*y *∈ *S*_*i *_: *G*_**w**_(**x**_*i*_, **y**_*i*_) <*G*_**w**_(**x**_*i*_, **y**).     (8)

In order to solve the reduced problem, we need to find the unknown weight vector **w **such that these constraints are satisfied. Again, since *G*_**w **_is a linear function, this set of constraints can translate into

∀*i*, ∀**y **∈ *S*_*i *_: ⟨**w**, ΔΨ_*i*_(**y**)⟩ > 0,     (9)

where ΔΨ_*i*_(**y**) = Ψ(**x**_*i*_, **y**) - Ψ(**x**_*i*_, **y**_*i*_). This reformulation of the constraints allows this problem to be solved in a much more elegant and computationally efficient manner. We use the powerful technique of Support Vector Machines to quickly determine the function *G*_**w**_, although many other techniques are possible.

### Iteratively constraining Support Vector Machines

Support Vector Machines (SVMs) are a fast and effective tool for generating functions from a set of labeled input training data. SVMs are able to determine a set of weights **w **for the function *G*_**w **_that will allow *G*_**w **_to accurately map all of the training example inputs **x**_*i *_to outputs **y**_*i*_. This problem can be formulated as a quadratic program, in which the variables are the weights **w **and a set of "slack variables" *ξ*_*i*_:

w^=arg⁡min⁡w12‖w‖2+Cn∑i=1nξi     (10a)
 MathType@MTEF@5@5@+=feaafiart1ev1aaatCvAUfKttLearuWrP9MDH5MBPbIqV92AaeXatLxBI9gBaebbnrfifHhDYfgasaacH8akY=wiFfYdH8Gipec8Eeeu0xXdbba9frFj0=OqFfea0dXdd9vqai=hGuQ8kuc9pgc9s8qqaq=dirpe0xb9q8qiLsFr0=vr0=vr0dc8meaabaqaciaacaGaaeqabaqabeGadaaakeaaieqacuWF3bWDgaqcaiabg2da9maaxababaGagiyyaeMaeiOCaiNaei4zaCMagiyBa0MaeiyAaKMaeiOBa4galeaacqWF3bWDaeqaaOWaaSaaaeaacqaIXaqmaeaacqaIYaGmaaWaauWaaeaacqWF3bWDaiaawMa7caGLkWoadaahaaWcbeqaaiabikdaYaaakiabgUcaRmaalaaabaGaem4qameabaGaemOBa4gaamaaqahabaacciGae4NVdG3aaSbaaSqaaiabdMgaPbqabaaabaGaemyAaKMaeyypa0JaeGymaedabaGaemOBa4ganiabggHiLdGccaWLjaGaaCzcamaabmaabaGaeGymaeJaeGimaadcbaGae0xyaegacaGLOaGaayzkaaaaaa@5480@

under the constraints

∀*i*, ∀**y **∈ *S*_*i *_: ⟨**w**, ΔΨ_*i*_(**y**)⟩ ≥ 1 - *ξ*_*i *_with ∀*i *: *ξ*_*i *_≥ 0.     (10b)

The only differences between these constraints and those in equation (9) is that (*i*) the strict inequality (> 0) is replaced by a non-strict inequality (≥ 1), and (*ii*) slack variables *ξ*_*i *_are introduced to allow a best-fit solution in the event of unsatisfiable constraints. The objective function minimizes the length of the weight vector (to normalize the constraints across various dimensions of **w**) and the size of the slack variables. The constant parameter *C *indicates how much a solution is penalized for violating a constraint. In practice, SVMs solve the dual of the minimization problem.

We can therefore use SVMs to determine our function *G*_**w**_; however, this only solves half of our problem.

Given a candidate *G*_**w **_we must then determine if equation (3) has been violated and add more constraints to it if necessary. To accomplish this task, we build off of work done by Tsochantaridis et al. [38] which tightly couples this constraint verification problem with the SVM **w **minimization problem.

First a loss function Δ(**y**_*i*_, **y**) is defined that weighs the goodness of the structures y^
 MathType@MTEF@5@5@+=feaafiart1ev1aaatCvAUfKttLearuWrP9MDH5MBPbIqV92AaeXatLxBI9gBaebbnrfifHhDYfgasaacH8akY=wiFfYdH8Gipec8Eeeu0xXdbba9frFj0=OqFfea0dXdd9vqai=hGuQ8kuc9pgc9s8qqaq=dirpe0xb9q8qiLsFr0=vr0=vr0dc8meaabaqaciaacaGaaeqabaqabeGadaaakeaaieqacuWF5bqEgaqcaaaa@2E3D@_*i*_. Smaller values of Δ(**y**_*i*_, **y**) indicate that structures **y**_*i *_and **y **are more similar. Adding this to the SVM constraints in equation (10b) gives

∀*i*, ∀**y **∈ *S*_*i *_: *ξ*_*i *_≥ Δ(**y**_*i*_, **y**) - ⟨**w**, ΔΨ_*i*_(**y**)⟩.     (11)

Using this we can decide when to add constraints to our reduced problem and which constraints to add. Since at every iteration of the algorithm we determine some **w **for the current *S*_*i*_, we can then find the value ξ^
 MathType@MTEF@5@5@+=feaafiart1ev1aaatCvAUfKttLearuWrP9MDH5MBPbIqV92AaeXatLxBI9gBaebbnrfifHhDYfgasaacH8akY=wiFfYdH8Gipec8Eeeu0xXdbba9frFj0=OqFfea0dXdd9vqai=hGuQ8kuc9pgc9s8qqaq=dirpe0xb9q8qiLsFr0=vr0=vr0dc8meaabaqaciaacaGaaeqabaqabeGadaaakeaaiiGacuWF+oaEgaqcaaaa@2E86@_*i *_assigned to variable *ξ*_*i *_as a result of the optimization. ξ^
 MathType@MTEF@5@5@+=feaafiart1ev1aaatCvAUfKttLearuWrP9MDH5MBPbIqV92AaeXatLxBI9gBaebbnrfifHhDYfgasaacH8akY=wiFfYdH8Gipec8Eeeu0xXdbba9frFj0=OqFfea0dXdd9vqai=hGuQ8kuc9pgc9s8qqaq=dirpe0xb9q8qiLsFr0=vr0=vr0dc8meaabaqaciaacaGaaeqabaqabeGadaaakeaaiiGacuWF+oaEgaqcaaaa@2E86@_*i *_corresponds to the "worst" prediction by **w **across the structures **y **∈ *S*_*i*_:

ξ^i=max⁡(0,max⁡y∈SiΔ(yi,y)−〈w,ΔΨi(y)〉).     (12)
 MathType@MTEF@5@5@+=feaafiart1ev1aaatCvAUfKttLearuWrP9MDH5MBPbIqV92AaeXatLxBI9gBaebbnrfifHhDYfgasaacH8akY=wiFfYdH8Gipec8Eeeu0xXdbba9frFj0=OqFfea0dXdd9vqai=hGuQ8kuc9pgc9s8qqaq=dirpe0xb9q8qiLsFr0=vr0=vr0dc8meaabaqaciaacaGaaeqabaqabeGadaaakeaaiiGacuWF+oaEgaqcamaaBaaaleaacqWGPbqAaeqaaOGaeyypa0JagiyBa0MaeiyyaeMaeiiEaGNaeiikaGIaeGimaaJaeiilaWYaaCbeaeaacyGGTbqBcqGGHbqycqGG4baEaSqaaGqabiab+Lha5jabgIGiolabdofatnaaBaaameaacqWGPbqAaeqaaaWcbeaakiabfs5aejabcIcaOiab+Lha5naaBaaaleaacqWGPbqAaeqaaOGaeiilaWIae4xEaKNaeiykaKIaeyOeI0YaaaWaaeaacqGF3bWDcqGGSaalcqqHuoarcqqHOoqwdaWgaaWcbaGaemyAaKgabeaakiabcIcaOiab+Lha5jabcMcaPaGaayzkJiaawQYiaiabcMcaPiabc6caUiaaxMaacaWLjaWaaeWaaeaacqaIXaqmcqaIYaGmaiaawIcacaGLPaaaaaa@5DB7@

This resulting ξ^
 MathType@MTEF@5@5@+=feaafiart1ev1aaatCvAUfKttLearuWrP9MDH5MBPbIqV92AaeXatLxBI9gBaebbnrfifHhDYfgasaacH8akY=wiFfYdH8Gipec8Eeeu0xXdbba9frFj0=OqFfea0dXdd9vqai=hGuQ8kuc9pgc9s8qqaq=dirpe0xb9q8qiLsFr0=vr0=vr0dc8meaabaqaciaacaGaaeqabaqabeGadaaakeaaiiGacuWF+oaEgaqcaaaa@2E86@_*i*_, which was determined using *S*_*i*_, can be compared to a similar ξ^′i
 MathType@MTEF@5@5@+=feaafiart1ev1aaatCvAUfKttLearuWrP9MDH5MBPbIqV92AaeXatLxBI9gBaebbnrfifHhDYfgasaacH8akY=wiFfYdH8Gipec8Eeeu0xXdbba9frFj0=OqFfea0dXdd9vqai=hGuQ8kuc9pgc9s8qqaq=dirpe0xb9q8qiLsFr0=vr0=vr0dc8meaabaqaciaacaGaaeqabaqabeGadaaakeaaiiGacuWF+oaEgaqcgaqbamaaBaaaleaacqWGPbqAaeqaaaaa@3018@ that is obtained by instead maximizing over Y
 MathType@MTEF@5@5@+=feaafiart1ev1aaatCvAUfKttLearuWrP9MDH5MBPbIqV92AaeXatLxBI9gBaebbnrfifHhDYfgasaacH8akY=wiFfYdH8Gipec8Eeeu0xXdbba9frFj0=OqFfea0dXdd9vqai=hGuQ8kuc9pgc9s8qqaq=dirpe0xb9q8qiLsFr0=vr0=vr0dc8meaabaqaciaacaGaaeqabaqabeGadaaakeaat0uy0HwzTfgDPnwy1egaryqtHrhAL1wy0L2yHvdaiqaacqWFyeFwaaa@3850@\{**y**_*i*_} in equation (12). This will tell us how much the constraints we are ignoring from Y
 MathType@MTEF@5@5@+=feaafiart1ev1aaatCvAUfKttLearuWrP9MDH5MBPbIqV92AaeXatLxBI9gBaebbnrfifHhDYfgasaacH8akY=wiFfYdH8Gipec8Eeeu0xXdbba9frFj0=OqFfea0dXdd9vqai=hGuQ8kuc9pgc9s8qqaq=dirpe0xb9q8qiLsFr0=vr0=vr0dc8meaabaqaciaacaGaaeqabaqabeGadaaakeaat0uy0HwzTfgDPnwy1egaryqtHrhAL1wy0L2yHvdaiqaacqWFyeFwaaa@3850@\{**y**_*i*_} will change the solution. The constraint that is most likely to change the solution is that which would have caused the greatest change to the slack variables. Therefore we would add the constraint to *S*_*i *_that corresponds to

y^′=arg⁡max⁡y∈Y\{yi}Δ(yi,y)−〈w,ΔΨi(y)〉.     (13)
 MathType@MTEF@5@5@+=feaafiart1ev1aaatCvAUfKttLearuWrP9MDH5MBPbIqV92AaeXatLxBI9gBaebbnrfifHhDYfgasaacH8akY=wiFfYdH8Gipec8Eeeu0xXdbba9frFj0=OqFfea0dXdd9vqai=hGuQ8kuc9pgc9s8qqaq=dirpe0xb9q8qiLsFr0=vr0=vr0dc8meaabaqaciaacaGaaeqabaqabeGadaaakeaaieqacuWF5bqEgaqcgaqbaiabg2da9maaxababaGagiyyaeMaeiOCaiNaei4zaCMagiyBa0MaeiyyaeMaeiiEaGhaleaacqWF5bqEcqGHiiIZt0uy0HwzTfgDPnwy1egaryqtHrhAL1wy0L2yHvdaiqaacqGFyeFwcqGGCbaxcqGG7bWEcqWF5bqEdaWgaaadbaGaemyAaKgabeaaliabc2ha9bqabaGccqqHuoarcqGGOaakcqWF5bqEdaWgaaWcbaGaemyAaKgabeaakiabcYcaSiab=Lha5jabcMcaPiabgkHiTmaaamaabaGae83DaCNaeiilaWIaeuiLdqKaeuiQdK1aaSbaaSqaaiabdMgaPbqabaGccqGGOaakcqWF5bqEcqGGPaqkaiaawMYicaGLQmcacqGGUaGlcaWLjaGaaCzcamaabmaabaGaeGymaeJaeG4mamdacaGLOaGaayzkaaaaaa@6878@

**Figure 5:**
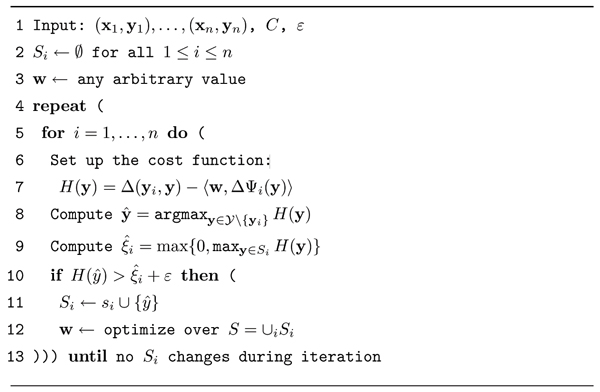
**Algorithm for iterative contraint based optimization**. Algorithm for iterative constraint based optimization.

Tsochantaridis et al. [38] show that by only adding constraints when y^
 MathType@MTEF@5@5@+=feaafiart1ev1aaatCvAUfKttLearuWrP9MDH5MBPbIqV92AaeXatLxBI9gBaebbnrfifHhDYfgasaacH8akY=wiFfYdH8Gipec8Eeeu0xXdbba9frFj0=OqFfea0dXdd9vqai=hGuQ8kuc9pgc9s8qqaq=dirpe0xb9q8qiLsFr0=vr0=vr0dc8meaabaqaciaacaGaaeqabaqabeGadaaakeaaieqacuWF5bqEgaqcaaaa@2E3D@' could change ξ^
 MathType@MTEF@5@5@+=feaafiart1ev1aaatCvAUfKttLearuWrP9MDH5MBPbIqV92AaeXatLxBI9gBaebbnrfifHhDYfgasaacH8akY=wiFfYdH8Gipec8Eeeu0xXdbba9frFj0=OqFfea0dXdd9vqai=hGuQ8kuc9pgc9s8qqaq=dirpe0xb9q8qiLsFr0=vr0=vr0dc8meaabaqaciaacaGaaeqabaqabeGadaaakeaaiiGacuWF+oaEgaqcaaaa@2E86@ by more than *ε*, one can attain a provable termination condition for the problem. The summary of this overall algorithm appears in Figure 5.

### Defining the set of valid structures

One final issue remains to be solved to complete our algorithm. We need to specify what Y
 MathType@MTEF@5@5@+=feaafiart1ev1aaatCvAUfKttLearuWrP9MDH5MBPbIqV92AaeXatLxBI9gBaebbnrfifHhDYfgasaacH8akY=wiFfYdH8Gipec8Eeeu0xXdbba9frFj0=OqFfea0dXdd9vqai=hGuQ8kuc9pgc9s8qqaq=dirpe0xb9q8qiLsFr0=vr0=vr0dc8meaabaqaciaacaGaaeqabaqabeGadaaakeaat0uy0HwzTfgDPnwy1egaryqtHrhAL1wy0L2yHvdaiqaacqWFyeFwaaa@3850@ and Ψ(**x**, **y**) are, and how to optimize *G*(**x**, **y**) over Y
 MathType@MTEF@5@5@+=feaafiart1ev1aaatCvAUfKttLearuWrP9MDH5MBPbIqV92AaeXatLxBI9gBaebbnrfifHhDYfgasaacH8akY=wiFfYdH8Gipec8Eeeu0xXdbba9frFj0=OqFfea0dXdd9vqai=hGuQ8kuc9pgc9s8qqaq=dirpe0xb9q8qiLsFr0=vr0=vr0dc8meaabaqaciaacaGaaeqabaqabeGadaaakeaat0uy0HwzTfgDPnwy1egaryqtHrhAL1wy0L2yHvdaiqaacqWFyeFwaaa@3850@. In general, Y
 MathType@MTEF@5@5@+=feaafiart1ev1aaatCvAUfKttLearuWrP9MDH5MBPbIqV92AaeXatLxBI9gBaebbnrfifHhDYfgasaacH8akY=wiFfYdH8Gipec8Eeeu0xXdbba9frFj0=OqFfea0dXdd9vqai=hGuQ8kuc9pgc9s8qqaq=dirpe0xb9q8qiLsFr0=vr0=vr0dc8meaabaqaciaacaGaaeqabaqabeGadaaakeaat0uy0HwzTfgDPnwy1egaryqtHrhAL1wy0L2yHvdaiqaacqWFyeFwaaa@3850@ can be exponentially large with respect to the sequence length, making brute-force optimization impractical. Our general approach is to structure Y
 MathType@MTEF@5@5@+=feaafiart1ev1aaatCvAUfKttLearuWrP9MDH5MBPbIqV92AaeXatLxBI9gBaebbnrfifHhDYfgasaacH8akY=wiFfYdH8Gipec8Eeeu0xXdbba9frFj0=OqFfea0dXdd9vqai=hGuQ8kuc9pgc9s8qqaq=dirpe0xb9q8qiLsFr0=vr0=vr0dc8meaabaqaciaacaGaaeqabaqabeGadaaakeaat0uy0HwzTfgDPnwy1egaryqtHrhAL1wy0L2yHvdaiqaacqWFyeFwaaa@3850@ and Ψ(**x**, **y**) in a way that allows optimization of *G*(**x**, **y**) through dynamic programming.

Most secondary-structure prediction tools use local features to predict which regions of a protein will be helical [2]. Individual residues can have propensities for being in a helix, they can act as helix nucleation sites, or they can interact with other nearby residues. This type of information can be well captured by Hidden Markov Models (HMMs). Equivalently, we choose to capture them using Finite State Machines (FSMs). The only difference between the FSMs we use and a non-stationary HMM is that the HMM deals with probabilities, which are multiplicative, while our FSMs deal with pseudo-energies, which are additive. To a logarithm, they are the same.

We define Y
 MathType@MTEF@5@5@+=feaafiart1ev1aaatCvAUfKttLearuWrP9MDH5MBPbIqV92AaeXatLxBI9gBaebbnrfifHhDYfgasaacH8akY=wiFfYdH8Gipec8Eeeu0xXdbba9frFj0=OqFfea0dXdd9vqai=hGuQ8kuc9pgc9s8qqaq=dirpe0xb9q8qiLsFr0=vr0=vr0dc8meaabaqaciaacaGaaeqabaqabeGadaaakeaat0uy0HwzTfgDPnwy1egaryqtHrhAL1wy0L2yHvdaiqaacqWFyeFwaaa@3850@ to be the language that is recognized by some FSM. Thus a structure **y **∈ Y
 MathType@MTEF@5@5@+=feaafiart1ev1aaatCvAUfKttLearuWrP9MDH5MBPbIqV92AaeXatLxBI9gBaebbnrfifHhDYfgasaacH8akY=wiFfYdH8Gipec8Eeeu0xXdbba9frFj0=OqFfea0dXdd9vqai=hGuQ8kuc9pgc9s8qqaq=dirpe0xb9q8qiLsFr0=vr0=vr0dc8meaabaqaciaacaGaaeqabaqabeGadaaakeaat0uy0HwzTfgDPnwy1egaryqtHrhAL1wy0L2yHvdaiqaacqWFyeFwaaa@3850@ will be a string over the input alphabet of the FSM. For example, that alphabet could be {*h*, *c*}, where *h *indicates that the residue at that position in the string is in a helix, and *c *indicates that it is in a coil region. A string **y **is read by an FSM one character at a time, inducing a specific set of transitions between internal states. Note that the FSMs we are considering do not need to be deterministic. However, they do need to satisfy the property that, for a given input string, there is at most one set of transitions leading from the initial state to a final state. We denote this sequence of transitions by *σ*(**y**) and note that *σ*(**y**) need not be defined for all **y**.

To define Ψ(**x**, **y**), we create a helper function *ψ*(**x**, *t*, *i*) which assigns a vector of feature values whenever transition *t *is taken at position *i *in the sequence **x**. For example, if a transition is taken to start a helix at position *i*, then *ψ*(**x**, *t*, *i*) might return features indicating that residues at position *i *- 3 to *i *+ 3 are associated with an N-terminal helix cap. The overall feature vector is the sum of these features across all positions in the sequence: Ψ(**x**, **y**) = ∑_*i *_*ψ*(**x**, *σ *(**y**)*i*, *i*).

The total cost *G*(**x**, **y**) follows the form of equation (7). We also specify an infinite cost for structures that are the wrong length or are rejected by the FSM:

G(x,y)={+∞if |x|≠|y| or σ(y)is undefined〈w,Ψ(x,y)〉otherwise     (14)
 MathType@MTEF@5@5@+=feaafiart1ev1aaatCvAUfKttLearuWrP9MDH5MBPbIqV92AaeXatLxBI9gBaebbnrfifHhDYfgasaacH8akY=wiFfYdH8Gipec8Eeeu0xXdbba9frFj0=OqFfea0dXdd9vqai=hGuQ8kuc9pgc9s8qqaq=dirpe0xb9q8qiLsFr0=vr0=vr0dc8meaabaqaciaacaGaaeqabaqabeGadaaakeaacqWGhbWrcqGGOaakieqacqWF4baEcqGGSaalcqWF5bqEcqGGPaqkcqGH9aqpdaGabaqaauaabaaaciaaaeaacqGHRaWkcqGHEisPaeaacqqGPbqAcqqGMbGzcqqGGaaidaabdaqaaiab=Hha4bGaay5bSlaawIa7aiabgcMi5oaaemaabaGae8xEaKhacaGLhWUaayjcSdGaeeiiaaIaee4Ba8MaeeOCaiNaeeiiaaccciGae43WdmNaeiikaGIae8xEaKNae8xkaKcccaGae0hiaaIaeeyAaKMaee4CamhcbiGaeWhiaaIaeeizaqMaeeyzauMaeeOzayMaeeyAaKMaeeOBa4MaeeyzauMaeeizaqgabaWaaaWaaeaacqWG3bWDcqGGSaalcqqHOoqwcqGGOaakcqWF4baEcqGGSaalcqWF5bqEcqGGPaqkaiaawMYicaGLQmcaaeaaieaacqWEVbWBcqWE0baDcqWEObaAcqWELbqzcqWEYbGCcqWE3bWDcqWEPbqAcqWEZbWCcqWELbqzaaaacaGL7baacaWLjaGaaCzcamaabmaabaGaeGymaeJaeGinaqdacaGLOaGaayzkaaaaaa@795F@

This cost is easy to optimize over Y
 MathType@MTEF@5@5@+=feaafiart1ev1aaatCvAUfKttLearuWrP9MDH5MBPbIqV92AaeXatLxBI9gBaebbnrfifHhDYfgasaacH8akY=wiFfYdH8Gipec8Eeeu0xXdbba9frFj0=OqFfea0dXdd9vqai=hGuQ8kuc9pgc9s8qqaq=dirpe0xb9q8qiLsFr0=vr0=vr0dc8meaabaqaciaacaGaaeqabaqabeGadaaakeaat0uy0HwzTfgDPnwy1egaryqtHrhAL1wy0L2yHvdaiqaacqWFyeFwaaa@3850@ by using the Viterbi algorithm. This algorithm proceeds in |**x**| rounds. In round *i*, the best path of length *s *starting from an initial state is calculated for each FSM state. These paths are computed by extending the best paths from the previous round by one transition, and picking the best resulting path for each state. The algorithmic complexity is *O*(|*FSM*|·|**x**|), where |*FSM*| is the number of states and transitions in the FSM.

### Implementation of the predictor

In our experiments, we have used an extremely simple finite state machine that is presented in Figure 1. Each state corresponds to being in a helix or coil region, and indicates how far into the region we are. States H4 and C3 correspond to helices and coils more than 4 and 3 residues long, respectively. Short coils are permitted, but helices shorter than 4 residues are not allowed, as even 3_10 _helices need at least 4 residues to complete one turn and form the first hydrogen bond.

Table 3 lists the basic features that were used in our experiments. These features can also be considered to be the parameters of our system, as our learning algorithm assigns an appropriate weight to each one. Our choice of features is motivated by observations that amino acids have varying propensities for appearing within an alpha helix as well as for appearing at the ends of a helix, an area termed the helix cap [39]. We introduce a single feature per residue to account for helix propensity, for a total of 20 parameters. For helix capping, we use a separate feature for each residue that appears at a given offset (-3 to +3) from a given end of the helix (N-terminal or C-terminal). This accounts for 20 * 7 * 2 = 280 parameters. Finally, we also introduce a feature for very short (2-residue) and short (3-residue) coils. Thus, there are a total of 302 parameters.

Table 4 illustrates how features are associated with the transitions of the FSM. This table corresponds to the *ψ *function described earlier; given an FSM transition and a position in the input sequence, it outputs a set of representative features. Most of this mapping is straightforward. In the case of helix caps (labels #1 and #2), features are emitted across a 7-residue window that is centered at position *n *- 1 (the previously processed residue).

None of the features we have used involve more than one residue in the sequence. We have experimented with more complicated cost functions that model pairwise interactions between nearby residues in a helix, namely between *n *and *n *+ 3 or *n *and *n *+ 4. So far we have not managed to improve our prediction accuracy using these interactions, possibly because each pairwise interaction adds 400 features to the cost function, leaving much room for over-learning. Indeed, with the expanded cost functions we observed improved predictions on the training proteins, but decreased performance on the test proteins.

We have also experimented with various loss functions Δ. We have tried a 0–1 loss function (0 unless both structures are identical), hamming distance (number of incorrectly predicted residues), and a modified hamming distance (residues are given more weight when they are farther from the helix-coil transitions). Each one gives results slightly better than the previous one.

## Competing interests

The authors declare that they have no competing interests.

## Authors' contributions

BG participated in the design of the method, implemented the bulk of the software, and drafted much of the manuscript. CWO participated in the design of the method, helped to implement the software, and drafted much of the manuscript. WT participated in the design of the method, helped to implement the software, and drafted much of the manuscript. AL refined the HMM cost models used for prediction. MvD participated in the design of the method and helped to draft the manuscript. SD initiated the project, participated in the design of the method, coordinated, and helped to draft the manuscript. All authors read and approved the final manuscript.

## Figures and Tables

**Table 1:** Number of parameters used for various protein structure predictors.

Number of Predictor Parameters		
**Category**	**Predictor**	**Number of Parameters**

Neural Net	PHD [9]	≥ 10,000
Neural Net	SSPro [23]	1400–2900
Neural Net	Riis & Krogh [10]	311–600
GOR + Linear Discrimination	DSC [6]	1000
HMM	Martin et al. [15]	471
HM-SVM	this paper (alpha only)	302

**Table 2:** Performance of our algorithm on all-alpha protein structure prediction.

SOV_*α *_and Q_*α *_Results					
Description	SOV_*α *_(%) (train)	SOV_*α *_(%) (test)	Q_*α *_(%) (train)	Q_*α *_(%) (test)	Training time (s)

Best run for SOV_*α*_	76.4	75.1	79.6	78.6	123
Average of 20 runs	75.1	73.4	79.1	77.6	162
Standard deviation of 20 runs	1.0	1.4	0.6	0.9	30

**Table 3:** Summary of basic features that are considered.

Features considered by predictor		
Name	Number of features	Description

*A*	1	Penalty for very short coil
*B*	1	Penalty for short coil
*H*_*R*_	20	Energy of residue *R *in a helix
CRi MathType@MTEF@5@5@+=feaafiart1ev1aaatCvAUfKttLearuWrP9MDH5MBPbIqV92AaeXatLxBI9gBaebbnrfifHhDYfgasaacH8akY=wiFfYdH8Gipec8Eeeu0xXdbba9frFj0=OqFfea0dXdd9vqai=hGuQ8kuc9pgc9s8qqaq=dirpe0xb9q8qiLsFr0=vr0=vr0dc8meaabaqaciaacaGaaeqabaqabeGadaaakeaacqWGdbWqdaqhaaWcbaGaemOuaifabaGaemyAaKgaaaaa@3070@	140	Energy of residue *R *at position i relative to C-cap
NRi MathType@MTEF@5@5@+=feaafiart1ev1aaatCvAUfKttLearuWrP9MDH5MBPbIqV92AaeXatLxBI9gBaebbnrfifHhDYfgasaacH8akY=wiFfYdH8Gipec8Eeeu0xXdbba9frFj0=OqFfea0dXdd9vqai=hGuQ8kuc9pgc9s8qqaq=dirpe0xb9q8qiLsFr0=vr0=vr0dc8meaabaqaciaacaGaaeqabaqabeGadaaakeaacqWGobGtdaqhaaWcbaGaemOuaifabaGaemyAaKgaaaaa@3086@	140	Energy of residue *R *at position i relative to N-cap

Total	302	

Each of these features corresponds to a parameter that is learned by our algorithm.

**Table 4:** Sets of features that are emitted by transitions in the FSM.

Sets of features emitted by FSM transition		
Label	Features	Description

#0	0	Coil defined as zero-energy
#1	∑i=−3+3CRn+i−1i−1 MathType@MTEF@5@5@+=feaafiart1ev1aaatCvAUfKttLearuWrP9MDH5MBPbIqV92AaeXatLxBI9gBaebbnrfifHhDYfgasaacH8akY=wiFfYdH8Gipec8Eeeu0xXdbba9frFj0=OqFfea0dXdd9vqai=hGuQ8kuc9pgc9s8qqaq=dirpe0xb9q8qiLsFr0=vr0=vr0dc8meaabaqaciaacaGaaeqabaqabeGadaaakeaadaaeWaqaaiabdoeadnaaDaaaleaacqWGsbGudaWgaaadbaGaemOBa4Maey4kaSIaemyAaKMaeyOeI0IaeGymaedabeaaaSqaaiabdMgaPjabgkHiTiabigdaXaaaaeaacqWGPbqAcqGH9aqpcqGHsislcqaIZaWmaeaacqGHRaWkcqaIZaWma0GaeyyeIuoaaaa@4013@	End of helix processing (C-cap)
#2	HRn+∑i=−3+3NRn+i−1i−1 MathType@MTEF@5@5@+=feaafiart1ev1aaatCvAUfKttLearuWrP9MDH5MBPbIqV92AaeXatLxBI9gBaebbnrfifHhDYfgasaacH8akY=wiFfYdH8Gipec8Eeeu0xXdbba9frFj0=OqFfea0dXdd9vqai=hGuQ8kuc9pgc9s8qqaq=dirpe0xb9q8qiLsFr0=vr0=vr0dc8meaabaqaciaacaGaaeqabaqabeGadaaakeaacqWGibasdaWgaaWcbaGaemOuai1aaSbaaWqaaiabd6gaUbqabaaaleqaaOGaey4kaSYaaabmaeaacqWGobGtdaqhaaWcbaGaemOuai1aaSbaaWqaaiabd6gaUjabgUcaRiabdMgaPjabgkHiTiabigdaXaqabaaaleaacqWGPbqAcqGHsislcqaIXaqmaaaabaGaemyAaKMaeyypa0JaeyOeI0IaeG4mamdabaGaey4kaSIaeG4mamdaniabggHiLdaaaa@4524@	Start of helix processing (N-cap)
#3	HRn MathType@MTEF@5@5@+=feaafiart1ev1aaatCvAUfKttLearuWrP9MDH5MBPbIqV92AaeXatLxBI9gBaebbnrfifHhDYfgasaacH8akY=wiFfYdH8Gipec8Eeeu0xXdbba9frFj0=OqFfea0dXdd9vqai=hGuQ8kuc9pgc9s8qqaq=dirpe0xb9q8qiLsFr0=vr0=vr0dc8meaabaqaciaacaGaaeqabaqabeGadaaakeaacqWGibasdaWgaaWcbaGaemOuai1aaSbaaWqaaiabd6gaUbqabaaaleqaaaaa@30BB@	Normal helix residue
#4	HRn MathType@MTEF@5@5@+=feaafiart1ev1aaatCvAUfKttLearuWrP9MDH5MBPbIqV92AaeXatLxBI9gBaebbnrfifHhDYfgasaacH8akY=wiFfYdH8Gipec8Eeeu0xXdbba9frFj0=OqFfea0dXdd9vqai=hGuQ8kuc9pgc9s8qqaq=dirpe0xb9q8qiLsFr0=vr0=vr0dc8meaabaqaciaacaGaaeqabaqabeGadaaakeaacqWGibasdaWgaaWcbaGaemOuai1aaSbaaWqaaiabd6gaUbqabaaaleqaaaaa@30BB@ + *A*	Helix after very short coil
#5	HRn MathType@MTEF@5@5@+=feaafiart1ev1aaatCvAUfKttLearuWrP9MDH5MBPbIqV92AaeXatLxBI9gBaebbnrfifHhDYfgasaacH8akY=wiFfYdH8Gipec8Eeeu0xXdbba9frFj0=OqFfea0dXdd9vqai=hGuQ8kuc9pgc9s8qqaq=dirpe0xb9q8qiLsFr0=vr0=vr0dc8meaabaqaciaacaGaaeqabaqabeGadaaakeaacqWGibasdaWgaaWcbaGaemOuai1aaSbaaWqaaiabd6gaUbqabaaaleqaaaaa@30BB@ + *B*	Helix after short coil

*R*_*i *_denotes the residue at position *i *in the protein, and *n *is the current position of the FSM.
